# New Perspective in the Formulation and Characterization of Didodecyldimethylammonium Bromide (DMAB) Stabilized Poly(Lactic-co-Glycolic Acid) (PLGA) Nanoparticles

**DOI:** 10.1371/journal.pone.0127532

**Published:** 2015-07-06

**Authors:** Rebecca Gossmann, Klaus Langer, Dennis Mulac

**Affiliations:** Institute of Pharmaceutical Technology and Biopharmacy, University of Muenster, Corrensstraße 48, 48149, Münster, Germany; University of Helsinki, FINLAND

## Abstract

Over the last few decades the establishment of nanoparticles as suitable drug carriers with the transport of drugs across biological barriers such as the gastrointestinal barrier moved into the focus of many research groups. Besides drug transport such carrier systems are well suited for the protection of drugs against enzymatic and chemical degradation. The preparation of biocompatible and biodegradable nanoparticles based on poly(lactic-co-glycolic acid) (PLGA) is intensively described in literature, while especially nanoparticles with cationic properties show a promising increased cellular uptake. This is due to the electrostatic interaction between the cationic surface and the negatively charged lipid membrane of the cells. Even though several studies achieved the successful preparation of nanoparticles stabilized with the cationic surfactants such as didodecyldimethylammonium bromide (DMAB), in most cases insufficient attention was paid to a precise analytical characterization of the nanoparticle system. The aim of the present work was to overcome this deficit by presenting a new perspective in the formulation and characterization of DMAB-stabilized PLGA nanoparticles. Therefore these nanoparticles were carefully examined with regard to particle diameter, zeta potential, the effect of variation in stabilizer concentration, residual DMAB content, and electrolyte stability. Without any steric stabilization, the DMAB-modified nanoparticles were sensitive to typical electrolyte concentrations of biological environments due to compression of the electrical double layer in conjunction with a decrease in zeta potential. To handle this problem, the present study proposed two modifications to enable electrolyte stability. Both polyvinyl alcohol (PVA) and polyethylene glycol (PEG) modified DMAB-PLGA-nanoparticles were stable during electrolyte addition. Furthermore, in contrast to unmodified DMAB-PLGA-nanoparticles and free DMAB, such modifications led to a lower cytotoxic activity against Caco-2 cells. In conclusion this study offers a closer and critical point of view on preparation, *in vitro* and analytical evaluation of DMAB-stabilized PLGA nanoparticles for the physiological use.

## Introduction

One of the greatest challenges of nanotechnology is the establishment of a suitable nanoparticulate carrier system for overcoming physiological barriers like the intestine. Oral administration is the preferred route of drug delivery because it provides the highest patient convenience and compliance [1, 2]. There is extensive literature concerning the correlation of oral drug absorption in humans and drug permeability across Caco-2 cell monolayers [3–5].

Especially in cancer research, many drugs show low bioavailability after peroral administration due to their poor stability, solubility, and permeability. Therefore, intravenous application in many cases is unavoidable. However, the encapsulation of such pharmaceutically challenging molecules within a polymeric nanoparticle matrix results in an increased drug absorption in targeted tissues or cells and protects the drug from enzymatic and hydrolytic degradation [6]. Consequently, the development of carrier systems could cause an increase in therapy efficiency and a decrease in negative side effects due to modified drug delivery [7].

Because of its proven biocompatibility and biodegradation, poly (DL-lactic-co-glycolic acid) (PLGA) is approved by the FDA for therapeutic use in humans and is one of the most successful starting materials for drug carrier preparations [8, 9]. PLGA possesses very low toxicity due to the fact that it undergoes hydrolysis to the monomers glycolic acid and lactic acid, which are endogenously metabolized in the human body using the Krebs cycle and eliminated as carbon dioxide and water [10, 11].

Basically nanoparticles formed of PLGA are prepared in the presence of polyvinyl alcohol (PVA) as a widely used steric stabilizer. The most described and applied preparation method is the emulsification-diffusion method, which leads to particle diameters in the range of 150 to 300 nm [12].

The degree of nanoparticle absorption by cells depends on diameter and surface properties such as surface charge or hydrophobicity, which in turn are associated with the strength of interaction between the nanoparticles and the cell membrane [13]. The surfactant used in nanoparticle preparation has a crucial influence on these factors. In the present study, the quaternary ammonium compound didodecyldimethylammonium bromide (DMAB) was used as a stabilizer because it leads to monodisperse nanoparticle preparations with a diameter of about 100 nm in combination with a stable positive surface charge. Preparing positively charged PLGA nanoparticles are described to improve cellular uptake and permeation over cellular barriers due to the fact that in contrast to PVA-stabilized systems adsorptive initiated endocytosis occurs increasingly [13–17].

Nevertheless recent studies illustrated the comparable pronounced cytotoxic activity of DMAB [18, 19], which is confirmed by our own cell viability screening. Therefore, the residual DMAB content of the formulation plays an important role for the characterization of the nanoparticle system, a fact that has received very little consideration in previous studies. The currently available instrumental methods for quantitation of quaternary ammonium surfactants are very expensive and time-consuming. Thus they are not easily useful in day-to-day laboratory work [20, 21]. Hence one goal of this study was to establish a DMAB quantification method, which not only serves its purpose perfectly in every laboratory but which also is a very cheap and fast alternative to the complex instrumental methods. Furthermore, we characterized DMAB-stabilized nanoparticles in due consideration of surfactant content, diameter, and zeta potential. Special attention was paid to stability under increasing electrolytic content.

This initial study took a critical look at the characterization and stability of DMAB-stabilized nanoparticles by physico-chemical as well as cell culture analysis. Two new modifications are proposed to minimize cytotoxicity and optimize electrolyte stability and therefore to enable nanoparticle application under physiological conditions in future experiments. These modifications could be helpful in preparing DMAB-stabilized nanoparticles for therapeutic use.

## Materials and Methods

### Materials

Poly (DL-lactide-co-glycolide) (PLGA) Resomer RG 502H and Poly(DL-lactide-co-glycolide)-co-polyethylene glycol diblock Resomer RGP d50155 were obtained from Evonik Industries AG (Darmstadt, Germany). Polyvinyl alcohol (PVA) 30,000–70,000 Da and didodecyldimethylammonium bromide (DMAB) were purchased from Sigma Aldrich (Steinheim, Germany).

For cell culture experiments Dulbecco’s modified Eagle’s medium (DMEM) and all used supplements were received from Biochrom AG (Berlin, Germany). VECTASHIELD Mounting Media with DAPI (4′,6-Diamidin-2-phenylindol) was purchased from Vector Laboratories Inc. (Burlingname, USA) and Wheat germ agglutinin (WGA) AlexaFluor 350 from Life Technologies (Carlsbad, USA). All other chemicals were obtained from Roth (Karlsruhe, Germany).

### Preparation of DMAB-stabilized nanoparticles

Nanoparticles were prepared by a literature described emulsification-diffusion method [14]. Briefly, 100 mg PLGA (Resomer RG 502H) were dissolved in 2.5 mL ethylacetate. The organic phase was added to a 5 mL aqueous solution containing 1.25, 2.5, 5, or 10 mg/mL DMAB followed by emulsification using a high-speed homogenizer at 15,000 rpm for 5 min. The resulting o/w emulsion was slowly poured into 5 mL water and stirred overnight at 550 rpm to remove the organic phase. The nanoparticles were washed with deionized water by threefold centrifugation (17,000 g, 25 min) and resuspension. This DMAB-stabilized nanoparticles are hereinafter referred to as PLGA-DMAB-NP.

### Physico-chemical characterization of DMAB-stabilized nanoparticles

The nanoparticles were evaluated for mean diameter and for polydispersity index (PDI), a dimensionless indicator of size distribution, by a Zetasizer (Nano ZS, Malvern Instruments, Malvern, UK) using the dynamic light-scattering technique. The zeta potential, representing both surface charge and stability, was also obtained by means of Zetasizer measurement based on electrophoretic mobility under an electric field.

The resulting particle yield after purification was determined gravimetrically. Therefore an aliquot (20.0 μl) of the respective nanoparticle sample was put in micro weighing dishes and dried for 2 h at 80°C until constant weight.

### Nanoparticle modifications

When using non-PEGylated PLGA as starting material, modification with PVA was performed by resuspension of the nanoparticle pellet after the third centrifugation step in aqueous PVA solution (1% w/v) followed by shaking overnight at 550 rpm. Afterwards, the nanoparticles were washed once with deionized water by centrifugation and resuspension (referred as PLGA-DMAB-PVA-NP).

PEG-modified DMAB nanoparticles were prepared similar to PLGA-DMAB-NP, but using PEGylated PLGA and emulsification for 10 min (PLGA-DMAB-PEG-NP).

### Influence of electrolytes on stability

The nanoparticles were titrated using a titration tool (Multi Purpose Titrator MPT-2, Malvern Instruments, Malvern, UK) with NaCl solution (250 mol/L) to evaluate the influence of electrolyte concentration on stability. Mean diameter, PDI, and zeta potential of triplicate experiments were determined by Zetasizer (Nano ZS, Malvern Instruments, Malvern, UK) measurements.

### Cell culture

Human epithelial colorectal adenocarcinoma cells (Caco-2) were commercially purchased from Institut für angewandte Zellkultur Dr. Toni Lindl GmbH (Munich, Germany).

The used cultivation medium of the investigated Caco-2 cells was Dulbecco’s modified Eagle’s medium (DMEM) with the addition of 1% (v/v) non-essential amino acids, 1% (v/v) penicillin/streptomycin/glutamine, and 10% fetal calf serum (FCS). The cells were subcultured twice a week with a ratio of 1:3, after reaching 80% confluence, or were used for experiments. Incubation experiments with nanoparticle formulations or free DMAB were performed using serum-free DMEM. For cultivation or incubation with substances, the cells were maintained at 37°C and 10% CO_2_.

### Evaluation of intracellular localization by fluorescence microscopy

Fluorescent nanoparticle formulations for localization experiments were prepared by dissolving 0.4 mg/mL of the fluorescence dye Lumogen F Red 305 in the ethyl acetate solution used during the nanoparticle preparation. Caco-2 cells were seeded with a density of 5 x 10^4^ cells/well on Millicell EZ slides (Merck KGaA, Darmstadt, Germany) and were allowed to grow overnight. This was followed by 4 h incubation at 37°C and 10% CO_2_ in serum free media with 0.02 mg/mL fluorescence dye-loaded PLGA-DMAB-NP, PLGA-DMAB-PVA-NP, and PLGA-DMAB-PEG-NP. Cells were washed once with phosphate buffered saline (PBS) and incubated with a Wheat germ agglutinin AlexaFluor 350 solution (10 μg/mL) for 10 min at room temperature for membrane staining. Cells were then washed three times with PBS, fixed in 4% para-formaldehyde for 15 min and washed again with PBS. For nuclear staining, cells were covered with VECTASHIELD Mounting Media with DAPI (Vector Laboratories Inc., Burlingname, USA). Samples were analyzed using a IX81 fluorescence microscope (Olympus, Hamburg, Germany) with filter systems including excitation at 360–370 nm, dichroic mirror at 400 nm, emission at 426–446 nm for DAPI and Alexa Fluor 350 and excitation at 535–555 nm, dichroic mirror at 565 nm, emission at 570–650 nm for Lumogen F Red 305.

### *In vitro* cell viability

The performed assay is based on the ability of living cells to reduce a water-soluble red dye, WST-1, to a yellow-colored formazan product by cellular dehydrogenases (Ishiyama et al., 1993). The nanoparticle formulations used for the study were freeze-dried and resuspended in serum-free DMEM.

Caco-2 cells were seeded in 96-well plates with a density of 1 x 10^4^ cells/well and incubated for 48 h. The cells were incubated with DMAB, PLGA-DMAB-NP, PLGA-DMAB-PVA-NP, and PLGA-DMAB-PEG-NP, respectively, in concentrations ranging between 0.5 and 2,000 μg/mL. After 24 h the cells were washed twice with PBS solution with calcium and magnesium and wells were filled with 100 μL serum-free medium.

Absorbance was measured at 460 nm using a microplate reader (Synergy Mx, BioTek Instruments, US) to obtain the background absorbance in order to minimize the error based on unspecific light scattering of the nanoparticles. Untreated cells were taken as negative control with 100% viability, and cells treated with DMSO were used as positive control with 0% viability.

After incubation with nanoparticle formulations or free DMAB for 24 hours 10 μL WST-1 were added to each well, followed by further incubation at 37°C for 1 h. EC_50_ values were calculated by curve fitting of the cell viability data finally measured at 460 nm (SigmaPlot version 11, Systat Software GmbH, Erkrath, Germany). Measurements were performed six fold in three passages and results are expressed as mean ± standard deviation (SD).

### Spectrophotometric assay of DMAB using bromothymol blue

The developed method to determine residual DMAB content in the nanoparticle suspension takes advantage of a direct spectrophotometric assay of quaternary ammonium compounds and is based on the ionic interaction between the cationic quaternary ammonium ion and the anionic dye bromothymol blue buffered at pH 7.5 [22]. Bromothymol blue solution is composed of 60 mg bromothymol blue dissolved in 100 mL of ethanol. Stock DMAB solution was prepared by dissolving an accurately weighed amount in water. Working DMAB solutions were prepared by diluting aliquots of the stock solution to concentrations between 0 and 300 μg/mL.

The assay was performed by mixing 160 μL of the nanoparticle dispersion with 25 μL NaOH (1 N). The mixture was incubated for 15 min at 60°C under shaking. Then 25 μL HCl, 140 μL bromothymol blue solution, and sufficient phosphate buffer pH 7.5 were added to a volume of 2.0 mL. The samples were shaken for 1 h at room temperature. After centrifugation at 20,000 g for 10 min to separate the supernatant containing excess bromothymol blue from the precipitated green DMAB-bromothymol blue complex, the supernatant was diluted with water at a ratio of 1:1 and measured photometrically at 610 nm. For linear assay calibration 160 μL DMAB working solutions (0 to 300 μg/mL DMAB) instead of the nanoparticle dispersion were used.

Validation of this method was performed according to the ICH harmonized tripartite guideline “Validation of analytical procedures: Text and Methodology Q2(R1)”. Therefore the method was tested with pure DMAB and DMAB plus hydrolyzed PLGA in three different DMAB concentrations covering the specified range for the procedure. The samples were measured six fold and analyzed as described above.

### Statistical methods

All investigations were done in triplicate. The results are presented as average value with standard deviation. In order to compare independent groups, one way ANOVA was performed using the software SigmaPlot version 11 (Systat Software GmbH, Erkrath, Germany). A significance level of ≤ 5% (p ≤ 0.05) between the groups was declared as a significant difference (*). With a significant level of ≤ 1% (p ≤ 0.01) the difference was defined as highly significant (**). For a most highly significant difference (***) the significance level was ≤ 0.1% (p ≤ 0.001).

## Results

### Preparation of DMAB-stabilized nanoparticles

For the preparation of DMAB-stabilized nanoparticles the method described by Bhardwaj et al. was adopted with suitable modifications [14]. In comparison to PVA-stabilized PLGA nanoparticles described earlier [23] the use of DMAB as a stabilizer led to smaller particles within a diameter range of about 70 nm to 110 nm in combination with a positive surface charge.

As seen in Table 1, an increased DMAB concentration from 2.5 to 10 mg/mL resulted in a significant decreasing particle diameter from about 110 nm to 70 nm. All of the nanoparticles were obtained as monodisperse samples with PDI values between 0.07 and 0.11. In contrast to the reduction of particle size the zeta potential increased from +26 mV to +46 mV with increasing DMAB concentration. The particles prepared with the lowest DMAB concentration of 1.25 mg/mL in the aqueous phase were not suitable for purification by centrifugation.

**Table 1:** Physicochemical properties of PLGA-DMAB-NP and PLGA-DMAB-PVA-NP as a function of the DMAB concentration of the aqueous phase.

	DMAB concentration of the aqueous phase [mg/mL]	Particle diameter [nm]	PDI	Zeta potential [mV]
PLGA-DMAB-NP	2.5	107.9 ± 2.9	0.11 ± 0.021	+ 26.3 ± 4.8
	5	93.5 ± 3.7	0.09 ± 0.010	+ 39.1 ± 0.5
	10	70.5 ± 1.8	0.07 ± 0.003	+ 46.3 ± 4.0
PLGA-DMAB-PVA-NP	2.5	136.3 ± 2.9	0.05 ± 0.016	+ 8.4 ± 2.5
	5	115.8 ± 4.1	0.09 ± 0.010	+ 22.4 ± 2.0
	10	95.5 ± 3.5	0.05 ± 0.017	+ 20.1 ± 1.0

Results are expressed as mean ± SD (n = 3).

### Nanoparticle modifications

In order to increase particle stability two different modifications of the preparation process were investigated: I. Resuspension of nanoparticles in PVA solution or II. The use of PLGA-PEG copolymer instead of the established PLGA polymer.

The analysis of PLGA-DMAB-PVA-NP showed, that adsorptive bound nonionic PVA led to a significant increase in particle diameter. However with a PDI below 0.1 the particle system remained monodisperse. The coating with PVA also resulted in a decrease of the zeta potential (Table 1).

At comparable DMAB concentrations the use of PLGA-PEG block copolymer as starting material led to an increasing diameter of about 100 nm and a PDI higher than 0.1. Only the application of 10 mg/mL DMAB in the aqueous phase resulted in acceptable nanoparticles with a diameter of 103.7 ± 2.4 nm and a PDI of 0.13. In contrast to nanoparticles based on PLGA the usage of the block copolymer led to a decreased zeta potential of +14 mV.

### Influence of electrolytes on colloidal stability

**Fig 1:**
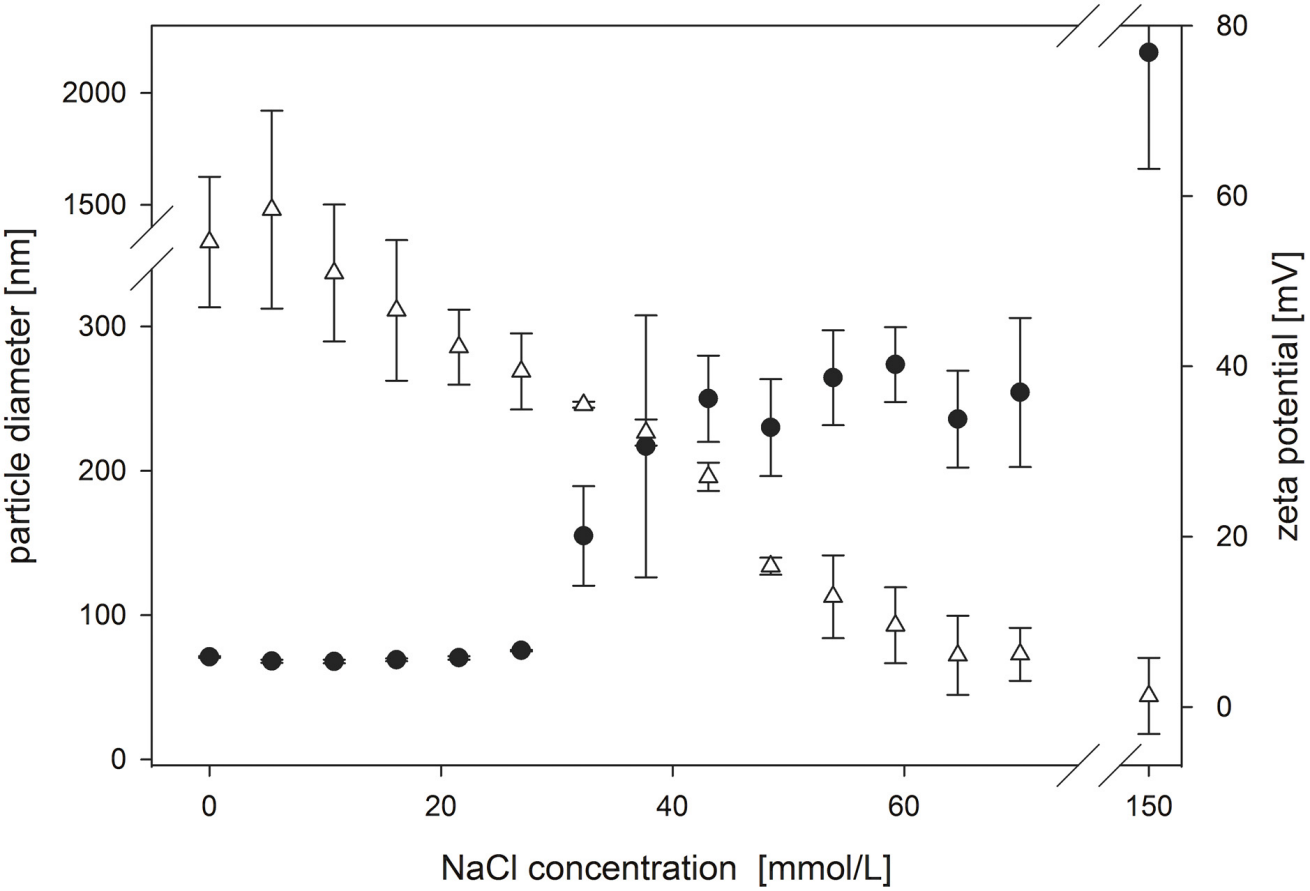
Electrolyte stability of PLGA-DMAB-NP. Titration of PLGA-DMAB-NP prepared at a DMAB concentration of 10 mg/ml and initial diameter of 70.5 nm ± 1.8 nm and zeta potential of +46.3 ± 4.0 mV with NaCl to 150 mM. Results are expressed as mean of triplicate experiments showing SD. ●Particle diameter; **Δ** Zeta potential.

As an electrolyte (NaCl) was added to the nanoparticle suspension, the magnitude of the zeta potential decreased progressively (Fig 1). This caused particle aggregation—as shown in an increase of the hydrodynamic diameter—of the nanoparticles from 70 nm to about 250 nm starting at 30 mM electrolyte concentration.

**Fig 2:**
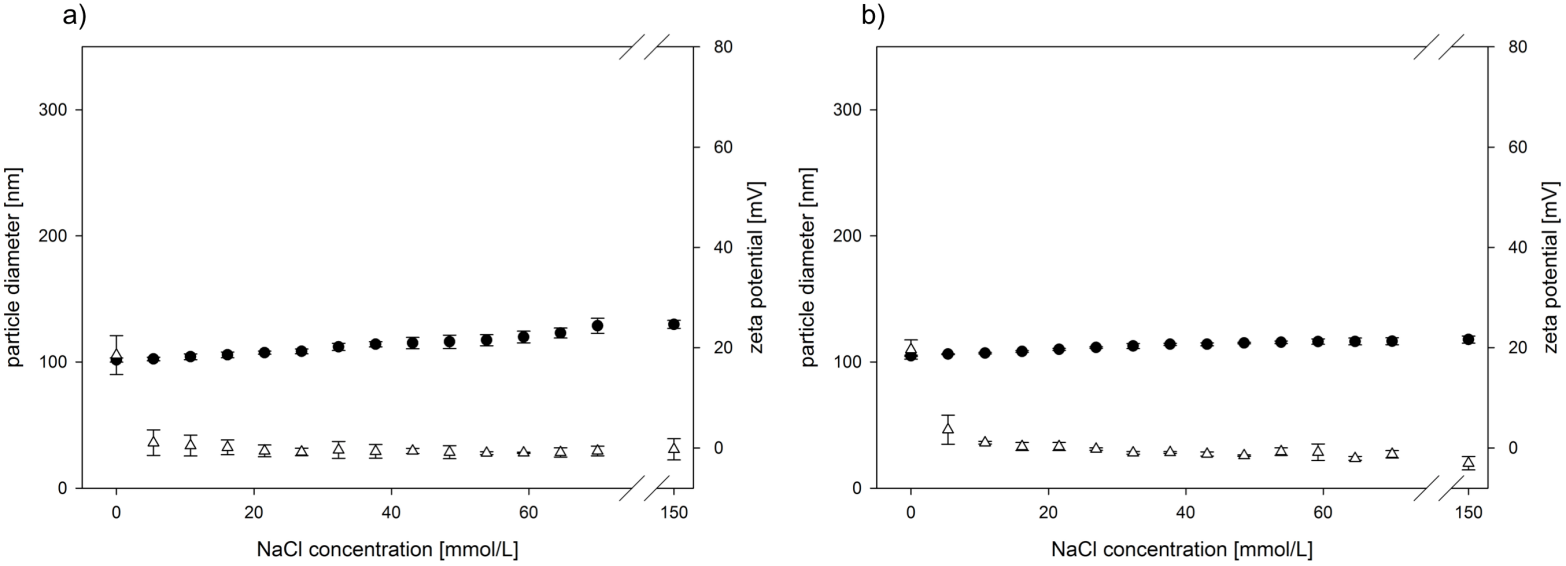
Electrolyte stability of modified NP. Titration of (a) PLGA-DMAB-PVA-NP (initial diameter of 95.5 ± 3.5 nm and zeta potential of +20.1 ± 1.0 mV) and (b) PLGA-DMAB-PEG-NP (initial diameter of 103.7 ± 2.3 nm and zeta potential of +14.0 ± 7.3 mV) prepared at a DMAB concentration of 10 mg/ml with NaCl to 150 mM. Results are expressed as mean of triplicate experiments showing SD. ●Particle diameter; **Δ** Zeta potential.

Even though the unmodified PLGA-DMAB-NP were unstable in electrolyte solutions, both the PLGA-DMAB-PVA-NP and PLGA-DMAB-PEG-NP remained stable under electrolyte influence. Although the zeta potential decreased rapidly to ±0 mV a constant diameter of about 110 nm was measured over the whole titration experiment. Taking a closer look at the diameter curve progression as a function of electrolyte concentration, it was striking to note that a small increase by about 10 nm occurred for both particle modifications (Fig 2).

### Evaluation of intracellular localization by fluorescence microscopy

**Fig 3:**
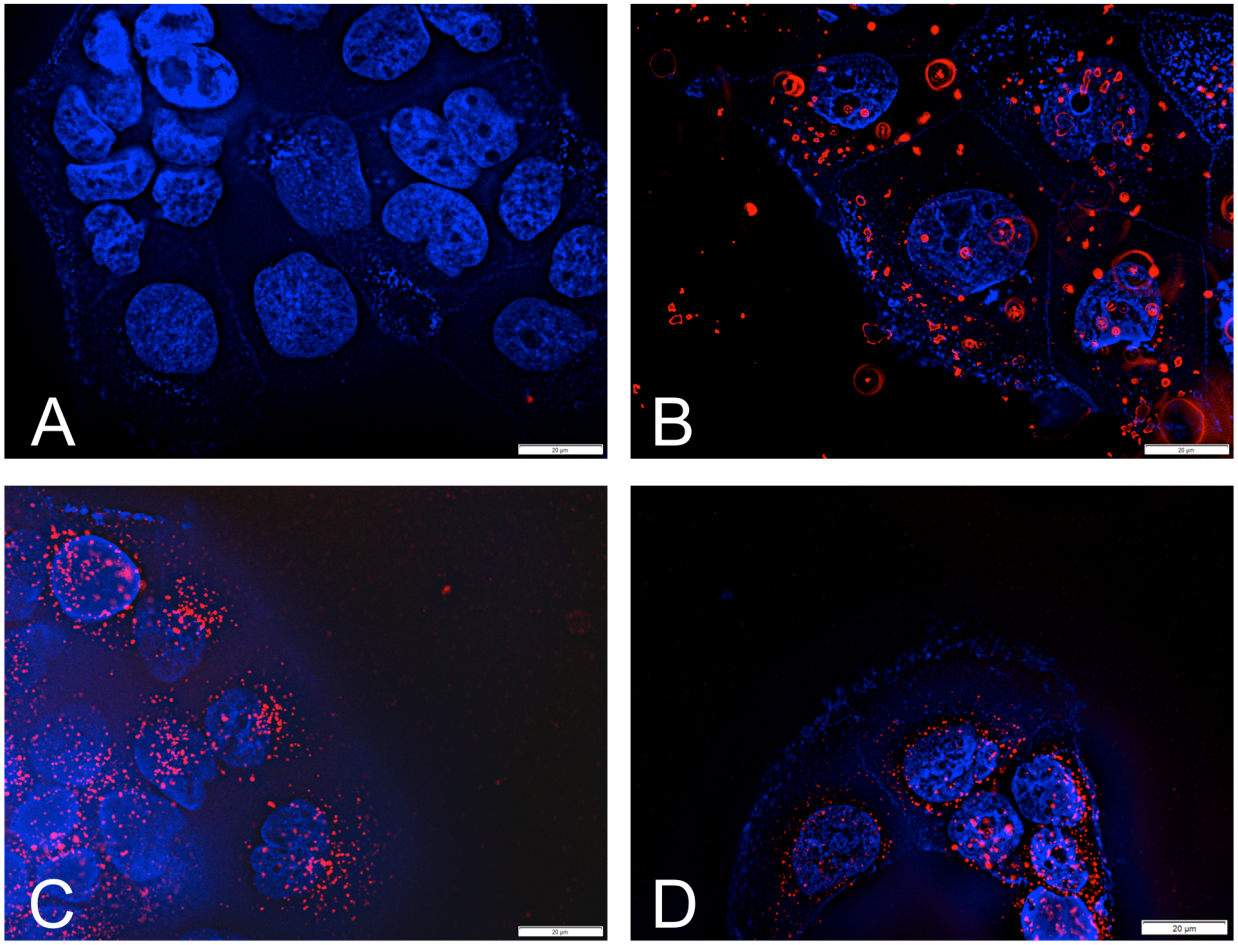
Evaluation of intracellular localization by fluorescence microscopy. Fluorescence visualization of nanoparticle formulations in Caco-2 cells: (A) control, (B) PLGA-DMAB-NP, (C) PLGA-DMAB-PVA-NP and (D) PLGA-DMAB-PEG-NP. Cell nuclei were stained with DAPI, membranes with WGA AlexaFluor 350. Nanoparticles are visualized due to incorporated fluorescence dye Lumogen F Red 305.

In the next step the ability of the nanocarriers to enter Caco-2 cells was examined by incubation with Wheat germ agglutinin AlexaFluor 350 and DAPI in the presence of the various dye-loaded NP formulations. Fig 3 illustrates that aggregates of unmodified PLGA-DMAB-NP adhere to the cell surface and could not be removed by repetitive washing steps. In contrast, the modified PLGA-DMAB-PVA-NP and PLGA-DMAB-PEG-NP did not aggregate and were internalized by the Caco-2 cells, therefore resulting in a high cellular uptake.

### In vitro cell viability

To evaluate the cytotoxic effect of the nanoparticles on human cells, Caco-2 cells were used as a model cell line. The effect of different nanoparticle formulations and free DMAB on cell viability was evaluated in a concentration range from 0.5–2000 μg/mL using the WST-1 assay.

Pure DMAB incubation resulted in a beginning decrease in cell viability above the concentration of 5 μg/mL and an EC_50_ value of 10 μg/mL. The PLGA-DMAB-NP exhibited a EC_50_ value of 50 μg/mL and an initial cytotoxicity at about 10 μg/mL (Table 2).

**Table 2:** EC_50_ values (μg/mL) of free DMAB, PLGA-DMAB-NP, PLGA-DMAB-PVA-NP, and PLGA-DMAB-PEG-NP in Caco-2 cells.

Composition	EC_50_ [μg/mL]
DMAB	10.6 ± 1.7
PLGA-DMAB-NP	54.8 ± 6.6
PLGA-DMAB-PVA-NP	350.3 ± 80.3
PLGA-DMAB-PEG-NP	996.5 ± 68.0

Results are expressed as mean ± SD (n = 18).

In contrast to the unmodified NP’s the EC_50_ values of PLGA-DMAB-PVA-NP and PLGA-DMAB-PEG-NP were 7–12 times higher, whereas first signs of cytotoxicity were observed at 100 μg/ml and 500 μg/mL for PLGA-DMAB-PVA-NP and PLGA-DMAB-PEG-NP, respectively. In all screenings the viability declined to 0% for the highest concentration. The EC_50_ values obtained showed that in contrast to the PLGA-DMAB-PVA-NP and PLGA-DMAB-PEG-NP unmodified PLGA-DMAB-NP were found to be cytotoxic to Caco-2 cells (Table 2).

### Spectrophotometric assay of DMAB using bromothymol blue

In consideration of the cytotoxic potential of pure or residual DMAB content in NP formulations, the need for a simple quantitation method is obvious. Because of the challenge to detect the non UV-active DMAB, the developed method takes advantage of the precipitation of a green complex of the anionic dye bromothymol blue in presence of a cationic quaternary ammonium compound.

Following the ICH guideline Q2(R1) the proposed method was validated with regard to linearity and accuracy of pure DMAB and DMAB in combination with hydrolyzed PLGA. To prove linearity a series of DMAB dilutions within the range of 0 and 300 μg/mL were analyzed as described above and a calibration curve was calculated with a correlation coefficient of 0.9984 (Table 3).

**Table 3:** Statistics of linear regression for the quantification of DMAB by spectrophotometry.

Parameter	Spectrophotometric calibration for DMAB
Slope b	-0.0025
Standard Error S_b_	3.597
Intercept a	1.0094
Standard Error S_a_	0.0070
Standard error of estimate S_y,x_	0.0108
Correlation coefficient r	0.9984
Number of samples	8

For verification of precision and accuracy test solutions containing DMAB and DMAB plus hydrolyzed PLGA at three different concentrations (low, medium, high) were prepared and measured in triplicate. Both the percentage of DMAB recovery and the confidential interval at a probability value of 5% were calculated (Table 4). At medium (150 μg/mL) and high (280 μg/mL) DMAB concentration and regardless of the presence of hydrolyzed PLGA the method showed a recovery in the range of 99.75–106.12%. in combination with small standard deviations as a measure of precision, whereas the method revealed no valid results at low concentration (50 μg/mL), which is expressed by the high standard deviation and 95% confidence interval (Table 4). This leads to the conclusion, that the sample solutions must be diluted in a way, that concentrations within the range of about 150 μg/mL and 280 μg/mL are achieved.

**Table 4:** Recovery data for the quantification of DMAB by spectrophotometry.

Parameter	DMAB			DMAB + hydrolyzed PLGA		
	level			level		
	Low	medium	high	low	medium	high
	(50 μg/mL)	(150 μg/mL)	(280 μg/mL)	(50 μg/mL)	(150 μg/mL)	(280 μg/mL)
Recovery [%]	102.93	106.12	101.59	95.10	99.75	101.68
S.D. [%]	30.85	3.23	0.96	17.22	1.05	2.61
Confidence interval (p = 95%)						
Upper limit [%]	179.57	114.13	103.98	137.87	102.36	108.16
Lower limit [%]	26.29	98.11	99.20	52.33	97.14	95.20
Maximum [%]	138.19	109.83	102.51	114.72	100.54	103.36
Minimum [%]	80.91	103.99	100.59	82.51	98.56	98.67
Number of samples	3	3	3	3	3	3

**Fig 4:**
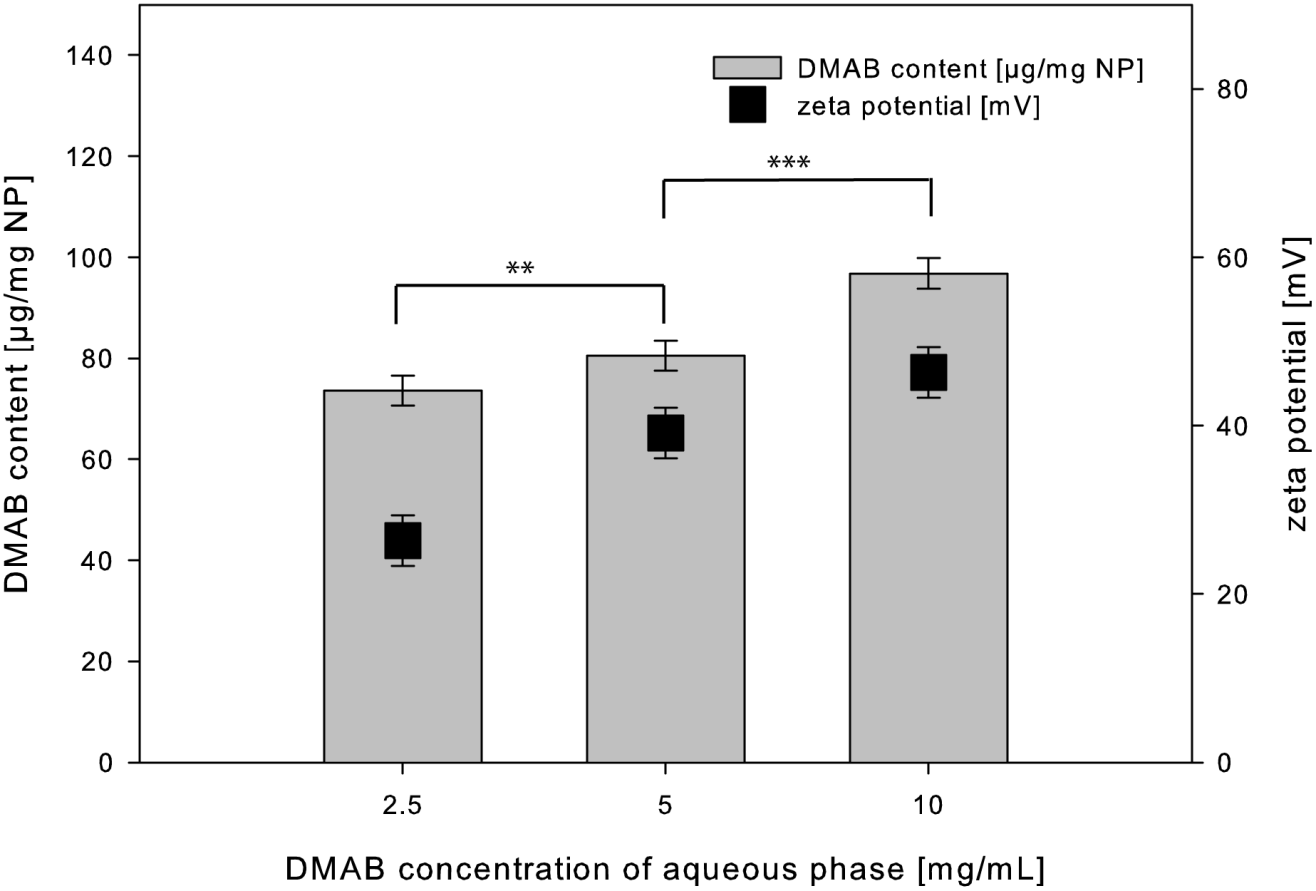
Relationship of DMAB content and zeta potential. Residual DMAB content and zeta potential after purification in relation to the used DMAB concentration of aqueous phase in particle preparation (mean ± SD, n = 3). The shown significances related to the particle diameter.

Based on the validated method the different nanoparticle formulations were analyzed for DMAB content after particle purification. The more DMAB was used for particle preparation the more DMAB remained in the particle suspension after purification, which is shown by Fig 4.

## Discussion

The present study was designed to reveal and overcome several problems in the preparation of DMAB-stabilized PLGA nanoparticles associated with agglomeration under raising electrolyte concentration. In principle, PLGA nanoparticles for drug delivery are routinely prepared with PVA as a stabilizer [24]. The resulting negative zeta potential leads to an electrostatic repulsion between the particle system and biological membranes with the consequence of a relatively low cellular uptake or transport over cellular barriers. DMAB is a quaternary ammonium compound with a smaller critical micelle concentration in comparison to PVA, in consequence smaller and positively charged nanoparticles result. The lowered particle diameter of about 100 nm as well as shift to a positive zeta potential leads to higher particle uptake in comparison to PVA stabilized nanoparticles as already described in literature [13–17, 25].

**Fig 5:**
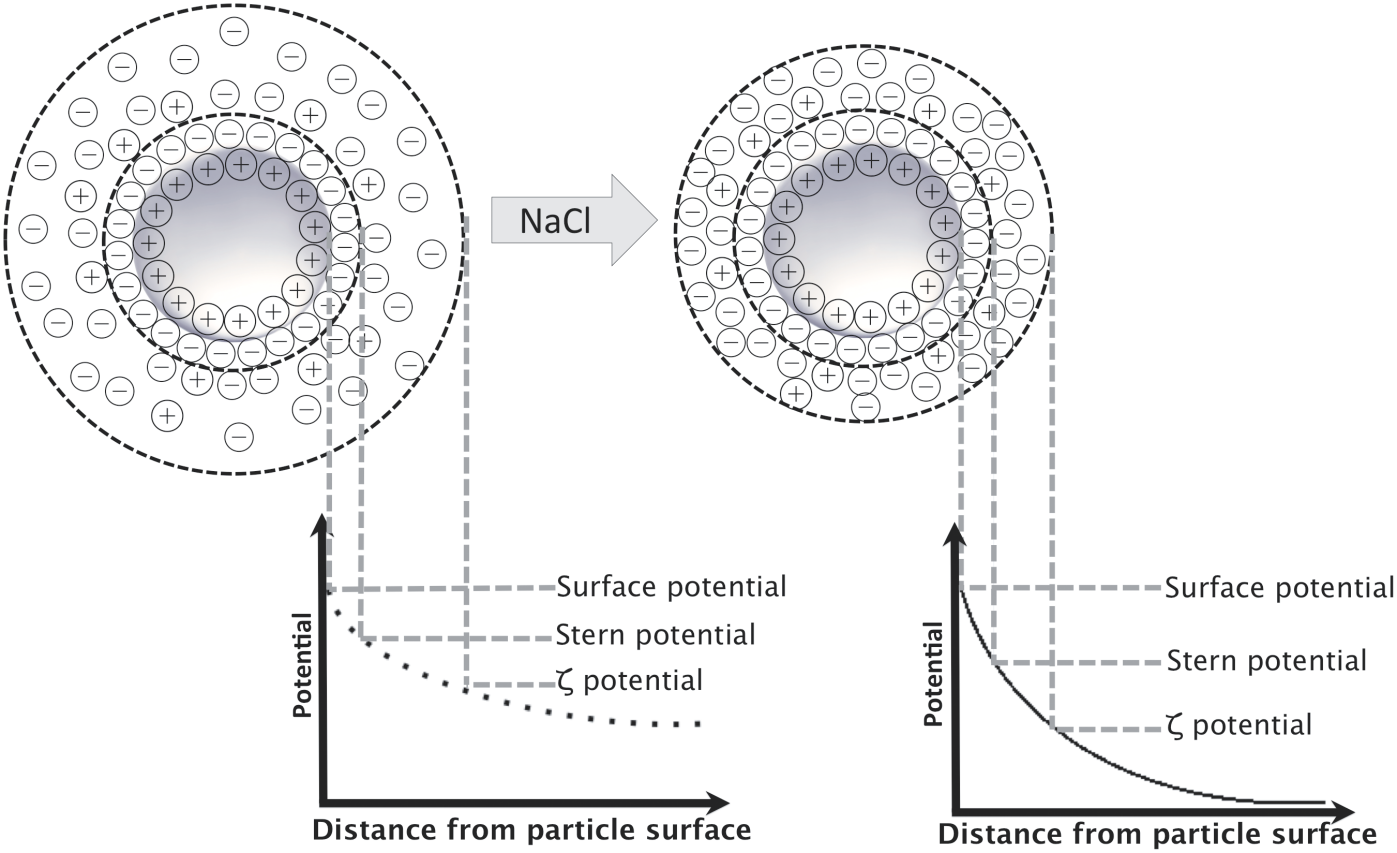
Schematic representation of electrolyte influence on electrical double layer and resultant zeta potential.

In a first step of the present study the influence of the used DMAB concentration on the physico-chemical parameters of the resulting nanoparticles was evaluated. During particle preparation the decreased interfacial tension as a function of the initial DMAB concentration led to smaller emulsion droplets in the primary emulsion during the homogenization step and to smaller nanoparticles after nanoprecipitation. The quantification of the residual DMAB content after purification showed that the more DMAB was initially used for particle preparation the more residual DMAB was quantified in the nanoparticles. Thus more DMAB remained at the interface between PLGA and water, which correlates with a higher surface charge and a higher zeta potential (Fig 5).

One of our aims in preparing DMAB-stabilized PLGA nanoparticles was to establish a nanoparticulate drug carrier system for future oral drug delivery. Therefore the application of a colloidal system in physiological media for use in cell culture studies or an in vivo set up requires electrolyte stability. Thus the smallest nanoparticles of about 70 nm in diameter, prepared with an initial DMAB concentration of 10 mg/mL in the aqueous phase, were titrated with an NaCl solution up to a final salt concentration of 150 mmol/L NaCl to evaluate the influence of electrolyte content on stability. For unmodified PLGA-DMAB-NP a rapid agglomeration even after the addition of minor NaCl concentrations was observed. The stability of the nanoparticle dispersions and their tendency to agglomerate below a zeta potential of +40 mV under electrolyte influence can be described within the context of attractive (such as van der Waals forces) and repulsive forces (such as electrostatic or steric forces) between particles by using the Derjaguin-Landau-Verwey-Overbeek (DLVO) theory [26]. The small molecule DMAB leads only to electrostatic but not steric stabilization depending on the thickness of the electrical double layer surrounding the particles. The first layer consists of fixed DMAB ions of the aqueous phase, whereas the second layer is a diffuse layer of nonfixed hydrated ions. Increasing electrolyte concentration results in a compression of the electrical double layer. As a result, the zeta potential decreases with increasing electrolyte concentration, even though the particle surface charge (inner Helmholtz plane) may be unchanged (Fig 5). Therefore, below a specific zeta potential, the repulsive forces are no longer sufficient to avoid particle agglomeration. This is confirmed by the observed agglomeration of PLGA-DMAB-NP after electrolyte addition. Therefore the suitability of unmodified DMAB-stabilized nanoparticles for physiological applications should be considered critical.

Consequently a steric stabilization of the particles besides pure electrostatic interaction is a rational approach. For this reason two modifications of the prepared PLGA-DMAB-NP were established to overcome the detected stability issues under electrolyte influence. On the one hand a PVA coating strategy was pursued and on the other hand the starting material PLGA was exchanged against a PLGA-PEG block copolymer. Both used hydrophilic compounds PEG and PVA provide steric stabilization of particle dispersions by a repulsion effect caused by a steric mechanism of stabilization involving both enthalpic and entropic contributions [27]. PEG, a linear polyether diol, forms highly hydrated polymer coils on the nanoparticle surface, which results in a low degree of immunogenicity and antigenicity [28]. In addition to steric stabilization, PEG modifications are known for a rather long time to prevent opsonization and represent the primary focus of the development of a long blood circulating drug carriers [29]. On the other hand PVA is the emulsifier most commonly used to stabilize PLGA nanoparticles. It attaches adsorptively on the particle surface and forms an interconnected network with the polymer at the interface [24]. The titration curves of the modified nanoparticle systems reveal comparable characteristics. The very first electrolyte application results in a breakdown of the zeta potential, whereas the nanoparticle diameter remains unaltered at about 100 nm, which is in contrast to unmodified PLGA-DMAB-NP (Fig 2). The observed slight increase in particle diameter as a function of electrolyte concentration is probably based on mechanisms of stabilization of hydrogen-bonding hydration through ionic hydration [30]. Therefore, addition of NaCl leads to a swelling of the polymer chains of PVA and PEG and to a slight increase in particle diameter without agglomeration.

The results of the electrolyte stability studies were supported by the cellular localization of the nanoparticles (Fig 3). Hence aggregates of the PLGA-DMAB-NP onto the cell surface were observed (Fig 3B), whereas both modified nanoparticle systems PLGA-DMAB-PVA-NP (Fig 3C) and PLGA-DMAB-PEG-NP (Fig 3D) showed a promising cellular internalization with a even distribution over the whole cell. The electrolytes present in cell culture medium led to an agglomeration of the non-steric stabilized PLGA-DMAB-NP and therefore to the observed bright spots. The pictures clearly underline the critical application of solely DMAB stabilized nanoparticle formulation under physiological conditions.

Furthermore in previous studies a high cytotoxic activity of DMAB for diverse cell types was demonstrated [18, 19]. This cytotoxic potential was confirmed by our cell culture results using a WST assay (EC_50_ = 10 μg/mL). Both free DMAB and PLGA-DMAB-NP showed a high cytotoxic activity against Caco-2 cells, as a common used model cell line to analyze gastrointestinal absorption. The parameter of cytotoxicity is crucial for further experiments regarding permeability of nanoparticles and therefore has to be screened for all preparations. The fact that PLGA-DMAB-NP showed high cytotoxic potential (EC_50_ = 50 μg/mL) though DMAB is not freely available may be attributed to the strong cationic charge on the particle surface. As previously described, cationic nanoparticles engage in strong ionic interactions with the negatively charged cell membranes [31]. In contrast, PLGA-DMAB-PVA-NP and PLGA-DMAB-PEG-NP are much less cytotoxic. Although further studies are required to identify mechanisms for the decreased cytotoxicity of PVA and PEG coated nanoparticles, the data collected suggest that the steric effect of both polymers shields the cells from direct contact to cytotoxic DMAB on the particle surface.

These results of cell-viability testing clarify the need for a quantitation method for the surfactant DMAB in particle systems. Described methods are not feasible without high technical complexity [20, 32]. Thus one challenge of this work was to establish and validate a simple and quick method for DMAB determination. This method takes advantage of a complex formation between the quaternary ammonium compound DMAB with the anionic dye bromothymol blue buffered at pH 7.5. The direct spectrophotometric assay was developed according to a previous described method of Lowry (1978) [22] with numerous modifications. In contrast to the previous study, the precipitated green complex of DMAB and bromothymol blue is separated from the supernatant containing the surplus bromothymol blue, which is measured spectrophotometrically at 610 nm. The results of the validation as described previously show that the method complies with ICH guidelines and thus is suitable for the determination of the DMAB content in solution and in nanoparticles after purification.

## Conclusion

DMAB is an appropriate surfactant for the preparation of small PLGA nanoparticles with a positive surface charge. However, upon closer examination of the stability under electrolyte influence, it becomes apparent that PLGA-DMAB-NP are possibly not qualified for application in physiological media. We have demonstrated that modification with hydrophilic polymers such as PVA or PEG leads to electrolyte stability and a decreased cytotoxicity against Caco-2 cells. Thus, modified DMAB nanoparticles could be useful in developing a suitable nanoparticulate drug carrier for biomedical applications in contrast to solely DMAB stabilized nanoparticle preparations.

This study also proposes a simple, suitable and convenient method for DMAB quantification that could be helpful in further studies for a comprehensive characterization of DMAB containing nanoparticle systems.
